# Myostatin: a multifunctional role in human female reproduction and fertility – a short review

**DOI:** 10.1186/s12958-022-00969-4

**Published:** 2022-07-02

**Authors:** Sijia Wang, Lanlan Fang, Luping Cong, Jacqueline Pui Wah Chung, Tin Chiu Li, David Yiu Leung Chan

**Affiliations:** 1 Assisted reproductive technologies unit, Department of Obstetrics and Gynecology, Faculty of Medicine, The Chinese University of Hong Kong, Hong Kong, 999077 SAR China; 2 Center for Reproductive Medicine, Henan Key Laboratory of Reproduction and Genetics, The First Affiliated Hospital of Zhengzhou University, Zhengzhou, 450003 China

## Abstract

Myostatin (MSTN) is member of the transforming growth factor β (TGF-β) superfamily and was originally identified in the musculoskeletal system as a negative regulator of skeletal muscle growth. The functional roles of MSTN outside of the musculoskeletal system have aroused researchers' interest in recent years, with an increasing number of studies being conducted in this area. Notably, the expression of *MSTN* and its potential activities in various reproductive organs, including the ovary, placenta, and uterus, have recently been examined. Numerous studies published in the last few years demonstrate that MSTN plays a critical role in human reproduction and fertility, including the regulation of follicular development, ovarian steroidogenesis, granule-cell proliferation, and oocyte maturation regulation. Furthermore, findings from clinical samples suggest that MSTN may play a key role in the pathogenesis of several reproductive disorders such as uterine myoma, preeclampsia (PE), ovary hyperstimulation syndrome (OHSS), and polycystic ovarian syndrome (PCOS). There is no comprehensive review regarding to MSTN related to the female reproductive system in the literature. This review serves as a summary of the genes in reproductive medicine and their potential influence. We summarized *MSTN* expression in different compartments of the female reproductive system. Subsequently, we discuss the role of MSTN in both physiological and several pathological conditions related to the female fertility and reproduction-related diseases.

## Introduction

Myostatin (MSTN), which is also called growth differentiation factor 8 (GDF8), was first reported by Alexandra McPherron and Se-Jin Lee in 1997. They discovered that MSTN is a negative regulator of skeletal muscle growth, and *Mstn* gene deletion could lead to hypermuscularity in mice [1]. A similar effect has been demonstrated in cattle, fish, sheep, dogs, and humans [2–6]. This gene was named Myostatin because it is a strong physiological regulator of muscle differentiation. MSTN is a member of the transforming growth factor-β (TGF-β) superfamily. The transforming growth factor-β (TGF-β) superfamily includes bone morphogenetic proteins (BMPs), growth differentiation factors (GDFs), TGF-βs, activins and inhibins, and anti-Müllerian hormone (AMH). The TGF-β superfamily plays an important functional role in physiology and pathology, such as the control of cell proliferation and differentiation, wound healing, the immune system, and skeletal diseases, fibrosis, and cancer [7–9].

Meanwhile, many factors in the TGF-β family, such as AMH, BMP15, and GDF9, are highly expressed and play an important role in the female reproductive system [10–12]. In recent years, many studies have shown that MSTN plays an essential role in the human female reproduction and fertility. The purpose of this review is to summarize the work to date on the expression and function of MSTN in the ovary, uterus and trophoblast. Furthermore, evidence from clinical samples has suggested that MSTN may have a role in the development of some reproductive diseases, such as uterine myoma, preeclampsia (PE), ovary hyperstimulation syndrome (OHSS), and polycystic ovarian syndrome (PCOS).

### Overview of MSTN

#### Gene mapping and expression of *MSTN*

*Mstn* was first identified in murine DNA using degenerate oligonucleotides corresponding to highly conserved sequences among TGF-β family members by PCR [1]. A murine skeletal muscle library was screened to obtain the complete cDNA sequence. In mice, the gene encoding *Mstn* is found on chromosome 1. In humans, the *MSTN* gene is located at position 32.2 on the long (q) arm of chromosome 2. The MSTN total molecular weight is 25.0 kDa, and all of the hallmarks of the TGF-β superfamily are present in the protein sequence, including a signal sequence for secretion, a proteolytic processing site, and a carboxy-terminal domain containing nine cysteine residues. MSTN is synthesized as a 376 amino acid precursor protein that includes a signal sequence, an N-terminal propeptide domain, and the C-terminal domain that gives rise to the active ligand, similar to other members of the TGF-β superfamily. The precursor protein of MSTN must be cleaved twice by proteolytic enzymes before it can be activated [13]. Furin family enzymes remove the 24-amino acid signal peptide during the first cleavage step [14]. The second cleavage by BMP1/Tolloid matrix metalloproteinase occurs at an RSRR (Arg-Ser-Arg-Arg) site at amino acids 240–243, numbered from the first amino acid following the signal sequence, and results in N-terminal and C-terminal domains of 27,640 Da and 12,400 Da, respectively [15]. Mature MSTN is a disulfide-linked dimer with a C-terminal domain identical in humans, mice, rats, pigs, chickens, turkeys, and dogs.

#### Expression and structure of MSTN in muscle

During the early stages of mouse embryogenesis, MSTN expression is localized to the myotome compartment of developing somites, and at later stages, MSTN expression is primarily expressed in skeletal muscle throughout the body [1]. *MSTN* mRNA or protein has been found in different organs and plasma in other animal species, according to several studies [16–18]. In humans, MSTN is primarily expressed in skeletal muscles, most prominently during the embryonic stage but also in maturity, and is considered to act as a negative regulator of muscular growth. MSTN is also produced in significant amounts in fat tissue [19] and the heart [20]. Low amounts of the *MSTN* gene are expressed in adipose tissue, while the protein MSTN is detectable in the blood [14]. A study on MSTN during lactation concerns the role of offspring serum MSTN levels on bone and negatively regulates muscle growth in the early postnatal period [21]. Myostatin null mice (*Mstn*^−/−^) demonstrate skeletal muscle fiber hyperplasia and hypertrophy [22] whereas *MSTN* deficits in larger mammals such as cattle and pigs engender muscle fiber hyperplasia [23, 24].

The mechanisms by which MSTN suppresses muscle development have been well studied. MSTN binds to the cell surface activin receptor type II or IIb (ActRII, ActRIIb) and recruits either Alk3 or Alk4 as a coreceptor [25]. This coreceptor, in turn, induces the phosphorylation of the SMAD family of transcription factors through the canonical TGF-β signaling pathway [26], although there is also evidence that MSTN can regulate muscle mass independently of SMAD signaling [27]. MSTN inhibits muscle stem cell proliferation [28] and differentiation [29], regardless of the pathway it takes, and it reduces the accumulation of adult muscle fiber protein [30], resulting in a loss of skeletal muscle mass [31, 32].

#### Function of MSTN

The principal role of MSTN is to regulate skeletal muscle development. However, the biological role of MSTN is not limited to inhibiting muscle development; it may have other redundant activities.

MSTN has a vital function in the heart, as demonstrated by a previous study, in maintaining cardiac energy homeostasis and the preventing of ventricular hypertrophy [33]. Cardiomyocytes in the peri-infarct area exhibit greater levels of the MSTN between 12 h and 30 days after induction of myocardial ischemia [20]. *MSTN* expression increases in individuals with decompensated heart failure [34] and congenital heart disease [35, 36]. Increased cardiac expression of BMP1 (which releases and activates myostatin from its latent complex), enhanced expression of the myostatin receptor ActRIIB, and finally increased SMAD2/3 activation all point to local effects of myostatin in the failing human heart [34]. Myostatin suppresses pathological hypertrophy in male mice stimulated with the α-adrenergic agonist phenylephrine, according to studies in myostatin knockout mice [37]. These findings indicate that the local effects of myostatin in the heart are moderate and stimulus dependent. MSTN protein levels rise rapidly after ischemia [38] and following aortic constriction(TAC)-induced hypertrophy in mice [39]. Genetically modified animals have revealed a different role in heart tissue [39–41]. In dystrophin-deficient animals, MSTN germ-line inactivation does not promote cardiac hypertrophy or reduce cardiac fibrosis, showing that MSTN does not operate in heart muscle as it does in skeletal muscle [42], demonstrating the significance of endogenous MSTN for adult cardiomyocyte metabolism [33].

MSTN is also associated with type 2 diabetes. Gene chip analysis found increased *MSTN* mRNA levels in skeletal muscle biopsy samples from individuals with type 2 diabetes and their nonobese but hyperinsulinemic relatives [43]. Exercise reduced muscle and plasma MSTN protein levels in insulin-resistant middle-aged men and thus lowered insulin sensitivity [44, 45]. Moreover, short-term inhibition of MSTN reduced insulin sensitivity in healthy male mice [44]. These findings show that MSTN expression may directly control skeletal muscle glucose absorption or utilization irrespective of muscle bulk. MSTN directly impact glucose absorption and utilization in cells. Furthermore, MSTN administration enhanced glucose absorption and glycolysis while decreasing glycogen production in cultured skeletal muscle cells [46]. Undoubtedly, these findings imply that MSTN may directly alter glucose absorption.

**Fig. 1:**
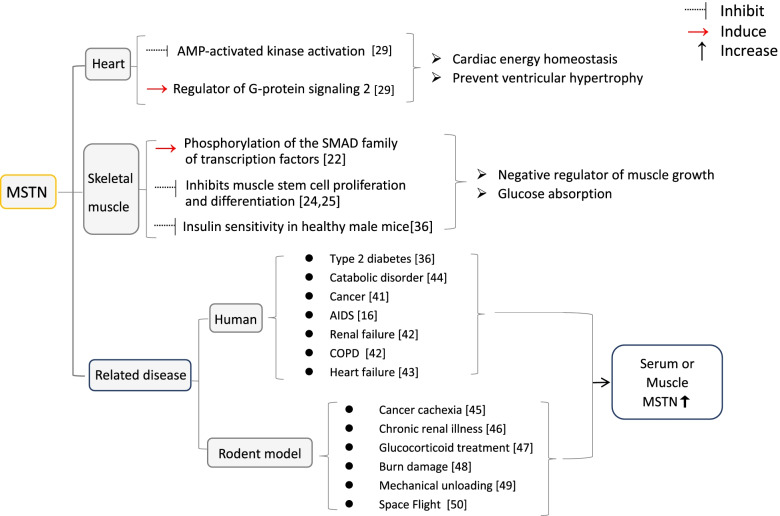
Summary of functions of MSTN

Patients with cancer [47], AIDS [16], renal failure, COPD [48], and heart failure [49] have elevated MSTN levels in their serum or increased MSTN expression in their muscles. Myostatin is involved in the response to these various disorders and may function as a possible regulator of increasing muscle atrophy in response to physiological and pathological stressors. MSTN levels are also elevated in elderly people and in those who have been on bed rest for an extended period of time [50]. Rodent models of cancer cachexia [51], chronic renal illness [52], glucocorticoid treatment [53], burn damage [54], mechanical unloading, and space flight all exhibit alterations [55, 56](Fig. 1).

### Distribution and functions in the female reproductive system

#### MSTN is involved in the regulation of ovarian function

Granulosa cells from small antral also expressed growth also known as GDF-8) that is a to be Previous research has shown that MSTN is expressed ubiquitously in numerous organs of chicken embryos, most notably the testis and ovary. MSTN may act as a growth regulator in the gonads during development [57]. A microarray study of granulosa cells (GCs) from various-sized antral follicles in bovine developing follicles identified *MSTN* mRNA [58]. Tsuchida discovered that *MSTN* was also expressed in human granulosa cells from small antral follicles [59]. Although *Mstn*knockout mice do not display infertility [60],, it is conceivable that compensatory growth factors, such as members of the TGF-β family members, mask essential functions or phenotypes in null mutant experiments. Similar discoveries have been made for other ovarian regulatory factors, such as LIF, which do not exhibit an ovarian null mutant phenotype but are known to influence germ cell development [61, 62]. However, simultaneously, a study on the fertility of myostatin gene mutant (*MSTN*^−/−^) pigs showed that although heterozygous *MSTN*^±^ sows were naturally reproductive, their litter size was significantly lower than that of wild-type (WT) sows, and the age at puberty was delayed in *MSTN*^±^ sows compared to WT sows [63], suggesting that the mstn gene has some effect on subfertility, but further studies on fertility in mice and humans are still needed. Many studies have shown that oocyte and theca cell-derived growth factors play essential roles in regulating ovarian functions [64–66].

##### In ovarian steroidogenesis

The central functional unit of the ovary is the ovarian follicle, which consists of one oocyte surrounded by granulosa and theca cells. Granulosa cells are required for normal oocyte development and steroid hormones synthesis. Theca cells are endocrine cells found in ovarian follicles that contribute significantly to fertility by generating the androgen substrate essential for ovarian estrogen production [67]. In 2015, Chan et al. demonstrated for the first time that MSTN is present in human granulosa cells and follicular fluid [68]. Another study discovered a negative correlation between MSTN and progesterone concentrations in human follicular fluid and the downregulation of steroidogenic acute regulatory (StAR) caused by MSTN through ALK5-mediated Smad3 and ERK1/2 signaling pathways in human granulosa cells [69]. StAR is the rate-limiting enzyme in the production of steroid hormones, and higher expression of aromatase and a decrease in StAR are associated with granulosa cell luteinization. Estrogen and progesterone are the two prominent ovarian steroids that regulate oocyte maturation and the cyclic changes occurring in the endometrium, respectively [70, 71]. A previous study found that MSTN increases estradiol production in granulosa cells by increasing cytochrome P450 aromatase (aromatase) expression and enhances the effects of follicle-stimulating hormone (FSH) by increasing FSH receptor levels while decreasing progesterone production and decreasing cell responsiveness to luteinizing hormone (LH) by decreasing LH receptor levels [72]. Further studies have shown that the effects of FSH on aromatase/estradiol induction were strengthened by pretreatment with MSTN for 24 h, whereas the effects of LH on StAR/progesterone stimulation were suppressed [72]. These studies suggest that MSTN may be a critical factor shaping cellular responses to gonadotropins and regulating ovarian steroid production. In 2018, Cheewasopit W. et al. also detected that MSTN is exists in bovine ovarian follicles and modulates granulosa and thecal steroidogenesis [73].

##### In the proliferation of granule cells

Granulosa cell proliferation and granulosa cell terminal differentiation are the two most significant processes during follicular development and are required for oocyte maturation, ovulation, and luteinization [74]. A wide range of regulatory endocrine and paracrine variables precisely control the functional transition of granulosa cells from a highly proliferative to a nonproliferative, terminally differentiated state throughout the periovulatory stage of the female reproductive cycle [75]. Previous research indicates that MSTN increases the expression of connective tissue growth factor (CTGF) via activin receptor-like kinase (ALK)4/5-mediated SMAD2/3-dependent signaling pathways [76]. CTGF is a multifunctional protein that belongs to the CCN family [77]. Many studies have indicated that CTGF plays critical roles in regulating numerous ovarian processes, including theca cell recruitment, follicular development, and corpus luteum vascularization in vivo and in vitro [78–81]. Furthermore, an increase in CTGF expression contributes to the MSTN-induced suppression of granulosa cell proliferation. The in vitro results demonstrate that MSTN and CTGF have negative regulatory functions in the regulation of proliferative events in human granulosa cells, which is consistent with previous findings [76].

##### In the extracellular matrix formation

Folliculogenesis is a highly regulated process in which multiple endocrine, paracrine, and autocrine hormones interact spatially and temporally to govern and organize the growth and development of the oocyte and its associated granulosa and theca cell layers. Intracellular communication among different cell types and the stroma is necessary for proper follicle growth and oocyte maturation. Thus, the extracellular matrix (ECM) within the follicle is thought to be critical in controlling follicular growth [82]. Lysyl oxidase (LOX), a critical enzyme in the final synthesis and stability of the extracellular matrix, is essential for follicle and oocyte maturation and is also necessary for sperm maturation [83]. Jiang et al. found that the level of LOX expression in the rat ovary is related to the quality of the oocytes produced [84]. Meanwhile, CTGF is a crucial mediator of extracellular matrix-related tissue remodeling in many organs, including the brain [85]. MSTN promotes CTGF production and secretion in human granulosa cells via SMAD2/SMAD3-SMAD4 dependent pathways mediated by the ALK5 type I receptor.

Furthermore, increased CTGF expression leads to the MSTN-induced elevation in LOX expression and activity. In vitro results demonstrate that MSTN and CTGF may play critical roles in regulating ECM and tissue remodeling during the periovulatory period [86]. Interestingly, these results oppose a previous study that found that MSTN signaling in mouse C2C12 myoblasts is dependent primarily on ALK4, but not ALK5, suggesting that type I receptor-mediated MSTN downstream signaling is cell-type specific [87].

##### In the regulation of oocyte maturation, cumulus oocyte complex expansion

The endocrinal interaction between the oocyte and its follicular somatic cells controls the growth of cumulus cells (CCs) during oocyte maturation, an essential stage in follicular development and eventual ovulation [88]. Furthermore, the quality of mature oocytes and subsequent developmental competence are connected to molecularly linked communications among mural granulosa cells, CCs, and oocytes [89–93]. During porcine IVM, MSTN supplementation enhanced mature oocyte quality by modulating phosphorylation of the p38 mitogen-activated protein kinase, as well as intracellular glutathione and reactive oxygen species levels [94]. The low-density lipoprotein (LDL) receptor to the steroidogenesis pathway was upregulated in CCs with higher oocyte maturity rates in the pregnancy group. A higher pregnancy rate is associated with the steroidogenesis signaling pathway by the LDL receptor in infertile women undergoing IVF procedures, according to the latest report [95]. StAR is crucial in the intracellular transport of cholesterol from the cytoplasm to the mitochondria in order to initiate steroidogenesis [96]. MSTN downregulates StAR in human granulosa cells [69], which may be associated with oocyte maturation. In conclusion, the expression level of the *MSTN* gene and the concentration of MSTN in the follicular fluid may be related to oocyte development and maturation; nevertheless, the mechanism of its role in regulating oocyte maturation needs to be investigated further.

The occurrence of the cumulus oocyte complex (COC) and the degree of cumulus expansion have been correlated with oocyte competence and could be used as signals for oocyte selection in an IVF program [97]. Pentraxin 3 (PTX3) is a member of the long pentraxin family and plays a critical role in female fertility [98]. PTX3 plays a vital role in the formation of extracellular matrix, which is essential for cumulus expansion, ovulation, and in vivo fertilization [98, 99], and *PTX3* expression levels in cumulus cells correlate with the quality and fertilization rates of the associated oocytes [100, 101]. The results of in vitro research and clinical data demonstrated a high association between PTX3 expression levels in cumulus cells and oocyte development and subsequent fertilization rates [101, 102]. MSTN inhibits PTX3 expression and secretion in human granulosa cells via a receptor complex composed of ACVR2A/ACVR2B type II receptors and ALK5 type I receptors, with SMAD-dependent signaling most likely involved [68]. These findings indicated that MSTN is physiologically involved in regulating follicular function and modulating COC expansion.

These studies suggest that MSTN is an intraovarian factor with a potential role in regulating ovarian processes in the human ovary. Any dysregulation or change in MSTN or its receptors may impact related intracellular pathways and influence ovarian functions, accounting for various reproductive diseases, including infertility. Understanding the normal and pathological role of intraovarian MSTN, particularly concerning granulosa cell functions and follicular fluid levels, may inform novel approaches to fertility regulation as well as enhance the diagnosis and treatment of ovarian diseases.

#### MSTN and ovarian disease

MSTN’s high level of expression and functions in the reproductive system suggest its potential importance in controlling female reproductive activities, particularly steroidogenesis, which is closely linked to pregnancy outcomes. Furthermore, data from clinical samples have highlighted the possible involvement of MSTN in the pathogenesis of certain ovary disorders, such as ovarian hyperstimulation syndrome (OHSS) and polycystic ovary syndrome ovaries (PCOS) [103–105].

##### MSTN and ovarian hyperstimulation syndrome

Ovarian hyperstimulation syndrome (OHSS) is one of the most common and potentially severe side effects of controlled ovarian stimulation (COS) in assisted reproductive technology (ART) [106]. OHSS occurs when ovarian enlargement secondary to follicular stimulation causes a shift of protein-rich fluid from intravascular to the third space [107]. This fluid shift is caused by increased vascular permeability in response to human chorionic gonadotropin activation (hCG) [107, 108]. To date, numerous risk factors have been related to the development of OHSS [109, 110]. High serum estradiol (E2) levels before hCG administration are considerably related to the development of OHSS. Furthermore, inhibiting E2 levels reduces the development of OHSS [111–113]. E2 plays essential roles in female reproduction [114]. The aromatase enzyme, encoded by the cytochrome P450 family 19 subfamily A member 1 (CYP19A1) gene, is widely known to play a pivotal role in the production of E2 [115]. According to the two-cell-two-gonadotropin theory, ovarian granulosa cells manufacture and generate E2 by converting theca cell-derived testosterone via aromatase [116]. MSTN treatment increases aromatase expression in human granulosa-lutein (hGL) cells, as previously stated [72]. In our most recent work, we demonstrated that MSTN stimulates aromatase expression and E2 synthesis in human granulosa cells via the ALK5 and SMAD2/3 signaling pathways. Furthermore, MSTN levels are increased in OHSS patients' follicular fluid and granulosa cells, leading to increased aromatase and E2 levels, both of which contribute to OHSS pathogenesis [104]. These findings may lead the way for developing of novel therapeutic approaches for OHSS in the future.

##### MSTN and polycystic ovary syndrome ovaries

Polycystic ovarian syndrome (PCOS) is a hormonal disorder that affects 5–10% of reproductive-age women and causes 75% of anovulatory infertility [117, 118]. PCOS is clinically defined by polycystic ovaries confirmed by ultrasonography, hyperandrogenism, and ovulatory dysfunction [119, 120]. To date, the specific cause of PCOS is unknown. Several genetic, hormonal, and environmental variables have been associated with the pathogenesis of PCOS [121, 122]. However, emerging data suggest that various intrinsic growth factors and their signaling are also implicated in establishing an altered intrafollicular milieu, leading to folliculogenesis abnormalities in PCOS [123–125]. PCOS follicle developmental abnormalities include disrupted steroidogenesis, aberrant follicle cell proliferation, and immature oocytes [124, 125]. A previous study showed that MSTN levels in the serum are higher in PCOS women than in non-PCOS women [126]. In addition, an immunohistochemical study demonstrated that MSTN was overexpressed in PCOS granulosa cells and large antral follicles. The increased expression of MSTN and its functional receptors (ACVR2A, ACVR2B, and ALK5) in PCOS antral follicles compared with normal ovary follicles suggests the potential for dysregulated MSTN in PCOS pathogenesis [126]. According to our prior study, MSTN levels in follicular fluid are higher in PCOS patients than in those without PCOS. High follicular MSTN levels cause reduced P4 production by enhancing ALK5-mediated downregulation of *StAR* expression in PCOS patients. These findings imply that MSTN suppresses P4 synthesis by downregulating *StAR* expression [127]. Intriguingly, a high level of MSTN is only detected in obese PCOS women, although there is no difference between nonobese women regardless of their PCOS condition [126]. Further research shows that MSTN therapy inhibits glucose metabolism in hGL cells. RNA-seq revealed that *MSTN* increased *SERPINE1* expression, which acted as a mediator in MSTN-induced glucose metabolism abnormalities. The SMAD2/3-SMAD4 signaling pathway is activated by MSTN to induce SERPINE1 expression and accumulation [128]. These studies contribute to better understanding of the role of MSTN in the pathophysiology of PCOS, which may lead to the development of different treatment strategies for clinical PCOS treatment.

#### MSTN and In-vitro Fertilization (IVF)

Since the start of in vitro* fertilization*/intracytoplasmic sperm injection-embryo transfer (IVF/ICSI-ET) for infertile patients, the most critical challenges have been practical prediction and improvement of clinical pregnancy results. Many studies have found that age, antral follicle count (AFC), serum hormone levels, recovered oocytes, and endometrial receptivity are effective clinical indicators for predicting pregnancy results in IVF/ICSI-ET patients [129–132]. Despite the parameters mentioned above, other researchers have found that various growth factors are significant in predicting ovarian response and pregnancy outcomes [133–137]. Members of the transforming growth factor superfamily (TGFs, bone morphogenetic proteins (BMPs), growth differentiation factors (GDFs), anti-Müllerian hormone (AMH), activins, and inhibins) are widely expressed in the ovary. They are involved in numerous aspects of female reproduction [138, 139]. TGF1 expression is considerably higher in pregnant women's follicular fluid than in nonpregnant women [140]. Recent research has established a strong correlation between serum AMH and ovarian response, retrieved oocyte number, and clinical pregnancy rates [141, 142]. During controlled ovarian hyperstimulation (COH), patients with the largest AMH drop have more retrieved oocytes and a higher clinical pregnancy rate [142]. A study revealed that serum MSTN protein levels varied dynamically during the COH process [103]. Serum MSTN levels were a reliable predictor of pregnancy in IVF/ICSI-ET patients. As we all know, serum hormone levels, particularly estradiol (E2) and progesterone (P4) fluctuate during the COH process and influence pregnancy outcomes in IVF/ICSI-ET patients [130, 143]. MSTN increases E2 levels while decreasing P4 levels in human granulosa cells, as previously stated [69, 72]. The concentration of MSTN was negatively correlated with the concentrations of LH and estradiol, as well as the number of antral follicles, according to the results of a clinical correlation analysis [144].

Many studies have suggested that MSTN may also be involved in embryo implantation. A study by Chun et al. on MSTN expression in the golden hamster uterus showed that exogenous MSTN could inhibit the growth of uterine smooth muscle cells (SMCs) and endometrial epithelial cells (EECs) in primary cultures. MSTN increased trophotoderm (TM) proliferation and hatching in cultured embryos but inhibited attachment [145]. This research reveals that MSTN can regulate preimplantation embryo activity. Meanwhile, higher levels of MSTN may be beneficial for pregnancy by maintaining a lower P4 level in blood before human chorionic gonadotrophin (hCG) administration; however, lower levels of MSTN may be critical for early embryo implantation by maintaining high P4 levels after hCG administration [103]. These results demonstrate that MSTN plays a critical role in ensuring a successful pregnancy, emphasizing the possible involvement of MSTN levels in ovarian response during the COH process.

#### MSTN and the placental

MSTN expression in the placenta has been observed in humans, mice, and rats [146–148]. In humans, MSTN is detected in the cytotrophoblast, syncytiotrophoblast, and extravillous trophoblast (EVT) cells of human placentas in the first trimester and term [149, 150]. Mitchell et al. discovered a negative association between placental MSTN and gestational age, as mature placentae had lower expression of both MSTN mRNA and protein than early gestation and preterm placental tissue [146].

##### MSTN involvement in placental function

Glucose is the primary form of carbohydrate delivered across the placenta and is a critical energy source for fetal development [151]. In human placental explants treated with MSTN, deoxyglucose absorption was increased, suggesting that MSTN participates in placental glucose homeostasis and may be a therapeutic target in conditions ranging from placental insufficiency to gestational diabetes [146]. MSTN is also detectable in the well-characterized model placental cell lines BeWo, JEG, and JAr [152]. Unlike Mitchell et al., Anthony et al. found that MSTN mainly inhibits glucose uptake into BeWo cells [152]. Anthony et al. used cell lines, but Mitchell et al. used primary tissue, which may explain the inconsistent results. In support of this finding, in primary isolated trophoblasts and cell lines, differences have been found in gene expression, methylation patterns, and the expression of HLA molecules [153–155].

In addition to its potential role in glucose uptake, MSTN may also affect the development of the placenta through interactions with cytokines. A series of cytokines, including tumor necrosis factor-α (TNFα), interleukin 6 (IL6), and IL1β, participate in the proliferation of cytotrophoblast cells in response to regulate both implantation and placental development [156, 157]. In *Mstn*^Ln/Ln^ mice lacking functioning MSTN, TNFα levels were shown to be lower in the plasma, but TNFα concentrations in the plasma increased after treatment with recombinant MSTN [158]. Treatment of C2C12 myotubes with recombinant MSTN resulted in enhanced IL6 mRNA and protein levels [159]. The capacity of MSTN to regulate cell growth has been used in the culture of human embryonic stem (HES) cells to maintain undifferentiated growth [160]. Trophoblastic cell proliferation, differentiation, and invasion are critical for placental development and function [1, 156]. In addition, these cells constitute the exchange surface between the maternal and fetal circulations.

In the HTR-8/SVneo cell line and in primary isolated EVT, MSTN had a positive impact on EVT proliferation and migration [149]. Follistatin-like 3 (*FSTL3*), also known as follistatin-related gene, is a regulatory glycoprotein that binds to members of the TGF superfamily, such as activins and MSTN and functions as an antagonist [161]. FSTL3 has a strong affinity for MSTN in particular [59]. The overall amount of FSTL3 in the human placenta is approximately 2–20 times higher than that in other organs, followed by the testes, heart, and pancreas [162]. In immortalized extravillous cytotrophoblast cells and primary extravillous cytotrophoblast cells derived from human first-trimester placentae, MSTN significantly upregulates the expression and synthesis of FSTL3 through the ALK5-SMAD2/3-mediated signaling pathway, further promoting cell invasiveness [163].

##### MSTN and placenta-related diseases

Many studies have shown that MSTN and FSTL3 may have a role in the regulation of normal placentation and preeclampsia. Maternal serum levels and placental expression of FSTL3 and MSTN were found to be considerably higher in women with preeclampsia [164–166]. Clinical samples revealed that FSTL3 levels were increased in the second trimester, which was related to an increased risk of developing preeclampsia [165]. Furthermore, FSTL3 expression is elevated in trophoblasts in response to hypoxia (a hallmark of preeclampsia), and FSTL3 deficiency impairs trophoblast invasion [167]. Another study found that variations in MSTN concentration in plasma can be detected early in preeclampsia and intrauterine growth restriction (IUGR) and PE-IUGR pregnancies, with increased expression of MSTN in presymptomatic PE plasma [168].

Because several studies have documented that MSTN is related to placental glucose homeostasis, as previously stated, MSTN may also affect the generation of gestational diabetes mellitus (GDM). GDM is characterized as carbohydrate intolerance leading to hyperglycemia of varying degrees of severity and affects 3–8% of pregnant women with onset or first detection during pregnancy [169, 170].A previous study discovered decreased maternal and placental FSTL3 concentrations, as well as unchanged maternal MSTN concentrations, in women with GDM compared to normal pregnancy, implying that decreased maternal and placental FSTL3 concentrations may play an important role in the pathogenesis of gestational diabetes mellitus [171]. The MSTN protein level (precursor and dimer) in the placentas of GDM and average glucose tolerant (NGT) pregnancies is significantly different. Furthermore, insulin treatment during GDM pregnancies increases the active form of MSTN and causes the placental expression to resemble that of NGT placentas. The MSTN level is considerably altered under the stress of obesity and impaired glucose metabolism reported in GDM [172].

#### MSTN and the uterus

MSTN-induced myometrial cell responsiveness and cell proliferation rates have been demonstrated in vitro. Simultaneously, the expression of MSTN in the uterus fluctuates throughout the estrous cycle and in response to steroid hormones, it has also been demonstrated in vivo [173]. Activin and MSTN are critical regulators of cell growth and differentiation and are members of the TGF-family of growth factors [174]. The ability of Activins to regulate FSH release from the anterior pituitary led to their discovery. Activins play a role in fibrosis, inflammation, and neurogenesis and are potent regulators of gonadal activities [175]. Activin A and MSTN have also been suggested to promote Smad7 expression in human myometrium explants. Both activin A and *MSTN* mRNAs were decreased following estradiol administration and remained unaltered following progesterone treatment but were increased in menopausal women compared to fertile women (proliferative phase) specimens. Activin A, *MSTN*, and follistatin-related genes were more abundant in human leiomyoma than in nearby human myometrium, although receptors, follistatin, and *Smad7* mRNAs remained unaltered [176]. These findings revealed that activin A and MSTN act on human myometrium, are steroid hormone-regulated and that the disruption of their signaling may contribute to fibroid growth. Additional studies suggest that activin A and MSTN act via the Smad signaling pathway on myometrial and leiomyoma cells. Their roles, however, are diverse in the two cell types as they exert cytostatic activity on healthy myometrial cells but only exert a fibrotic effect on leiomyoma cells [172]. These findings contribute to a better understanding of the modifications that occur in leiomyoma.

Furthermore, activin A and MSTN inhibited cell proliferation in primary myometrial cells but not in leiomyoma cells and decreased expression of proliferating cell nuclear antigen and Ki67 in myometrial cells was also detected. However, in both myometrial and leiomyoma cells, activin A and MSTN increase Smad2/3 signaling but do not influence ERK or p38 signaling, suggesting that activin A and MSTN can have antiproliferative and/or fibrotic effects on both cell types via Smad2/3 signaling [177]. In the human endometrium, activin A plays a critical role in the menstrual cycle, with higher expression during the late secretory phase and decidualization [178, 179]. Follistatin is a binding protein that increases muscle growth by directly blocking MSTN [180]. Follistatin also participates in the inflammatory response by inhibiting activin and bone morphogenetic protein signaling, and it is upregulated during endothelial cell proliferation and migration [181, 182]. Follistatin expression is abnormal in endometrioma and eutopic endometria of women with endometriosis [183, 184], implying that failure of this pathway may contribute to endometriosis-related infertility. Patrizia Carrarelli et al. documented that adenomyotic tissues express significant quantities of MSTN, follistatin, and activin A, all of which have a role in proliferation, apoptosis, and angiogenesis. Increased expression of their receptors supports the concept that these growth factors may exert a local effect in adenomyosis. The increased expression of ActRIIa, ActRIIb, and follistatin in the endometrium of these patients may contribute to their infertility due to adenomyosis [185] (Table 1).

**Table 1:** Effects of MSTN in the female reproductive system

Function			Model	Treatment	Main Results (Ref.)
**Ovary**	Physiological	Ovarian steroidogenesis	Human granulosa cells	SVOG cells: 30 ng/mL rhMSTN	Steroidogenic acute regulatory ↓ [69]
				hGL cells: recombinant human MSTN	Estradiol production ↑ [72]
					Cytochrome P450 aromatase ↑ [72]
					FSH receptor levels ↑ [72]
					Progesterone production ↓ [72]
					Cell responsiveness to luteinizing hormone ↓ [72]
					LH receptor levels ↓ [72]
			Bovine granulosa cells	Granulosa: MSTN (100 ng/ml)	Basal CYP19A1 expression and estradiol secretion ↑ [73]
					Cell number ↑ [73]
					Basal and FSH-induced HSD3B1 expression and progesterone secretion ↓ [73]
			Bovine theca cells	Theca cells: MSTN (100 ng/ml)	Basal and LH-stimulated androgen secretion ↓ [73]
		Proliferation of granule-cell	Human granulosa cells	SVOG cells: 30 ng/ml recombinant human MSTN	Expression of CTGF ↑ [76]
					Granulosa cell proliferation ↓ [76]
		ECM formation	Human granulosa cells	SVOG cells: MSTN (30 ng/ml)	LOX expression and activity ↑ [86]
				SVOG cells: MSTN (1/10/100 ng/ml)	Secretion of CTGF ↑ [86]
		Oocyte maturation	Porcine oocyte	During the entire IVM of COCs: 1.3 ng/mL MSTN	Mature oocyte quality ↑ [94]
					Modulate phosphorylation of the p38 mitogen-activated protein kinase [94]
					Modulate intracellular glutathione and ROS levels [94]
		COC expansion	Human granulosa cells	SVOG cells: recombinant human MSTN (1/10/ 100 ng/ml)	PTX3 expression and secretion ↓ [68]
	Pathological	OHSS	Human granulosa- lutein cells	hGL cells: MSTN (100 ng/mL)	Aromatase expression ↑ [104]
			Human follicular fluid	OHSS patients' follicular fluid and granulosa cells	MSTN level ↑ [104]
		PCOS	Human	PCOS patients’ granulosa cells	MSTN level ↑ [186]
				PCOS patients’ serum and follicular fluid	MSTN level↑ [127]
**Placenta**	Physiological	Glucose uptake	Human placental explants	Human term placental explants: MSTN (1 mg/ml)	Deoxyglucose absorption ↑ [146]
			Placental cell lines (BeWo)	BeWo cells: MSTN (0.2 nM /0.4 nM/4 nM /40 nM)	Glucose uptake in Bewo Cells ↓ [152]
		Placenta development	Mice model	Mstn^Ln^/^Ln^ mice treated with recombinant MSTN	TNFα concentrations in plasma ↑ [158]
				C2C12 myotubes treated with recombinant MSTN	IL6 expression ↑ [159]
			Human embryonic stem cells	hESC cultured with 20 ng/mL MSTN	Maintain undifferentiated growth of Hes Cells [160]
		Cell invasiveness	Human trophoblasts	EVT and HTR8/SVneo cells cultured with MSTN (25 ng/mL)	Expression and synthesis of FSTL3 ↑ [163]
					Cell invasiveness ↑ [163]
	Pathological	Pre-eclampsia	Human	Pre-eclamptic women’s serum	MSTN in women with pre-eclampsia ↑ [164]
		IUGR	Human	Plasma of Presymptomatic Women	Myostatin dimer in placentae ↑ [168]
		GDM	Human	Serum of women with GDM	Placental FSTL3 in women with GDM ↓ [171]
					Unchanged MSTN Concentration [171]
**Uterus**	Physiological	Myometrial cell	Pregnant human myometrial 1 cell line	PHM1 cell line supplemented with 1/10 nM MSTN	Myometrial cell growth ↓ [173]
			Human	Adjacent normal myometrium	Activin a and MSTN exert cytostatic activity on healthy myometrial cells [176]
	Pathological	Leiomyoma	Human	Fibroid from women undergoing hysterectomy	Activin a, MSTN, and follistatin-related genes in human leiomyoma ↑ [176]
		Endometriosis	Human	Serum from women with endometriosis	Follistatin level ↑ [183]
		Adenomyosis	Human tissue	Adenomyotic tissues from women undergoing hysterectomy	MSTN, follistatin, and Activin a in adenomyotic tissues ↑ [185]

## Conclusion

MSTN has been detected in a range of human tissues, including muscle, plasma, fat tissue, heart, adipose tissue, and the lung, where it has various of functions. However, an accumulating number of publications have revealed that MSTN is expressed in human reproductive systems and performs different roles. Mature MSTN proteins have been identified in follicular fluid, indicating that the protein is functional. According to numerous functional investigations, MSTN is involved in the regulation of steroidogenesis, gonadotrophin responsiveness, cell proliferation, LOX expression, LOX activity, and PTX3 expression in human germ cells. These findings suggest that MSTN may play a critical role in influencing the final differentiation processes in emerging follicles, most likely by acting as a maturation stimulant at the cellular and subcellular levels. MSTN is found in the human placenta and uterus, where it participates in a range of physiological and pathological processes. Adenomyosis, PCOS, and OHSS are just a few of the reproductive ailments that have been connected to MSTN. MSTN is involved in the regulation of oocyte maturation, and the addition of exogenous MSTN to the culture medium promotes the maturation of porcine oocytes, but as MSTN is expressed in several tissues, treatment with exogenous MSTN may lead to inhibition of oocyte expansion in the ovary, affecting the secretion of steroid hormones such as E2 and progesterone, and may promote the development of OHSS, leading to metabolic disorders in PCOS patients. However, notably, that treatment with exogenous MSTN may also have effects on organs and tissues outside the reproductive system, such as muscle wasting and myocardial fibroblast hypertrophy.

In addition, there are many unanswered questions about MSTN's role in the female reproductive system. More research is needed to determine the relationship between the pathophysiology of these disorders and MSTN, as well as the molecular mechanisms implicated, such as, the dynamic changes that occur prior to and after embryo implantation, the concentration of MSTN that is most conducive to human embryonic development, the role of MSTN in the regulation of the human embryo and the molecular mechanisms that regulate it; the regulatory mechanisms of MSTN for ovarian development and the effects of steroid-hormone interactions on IVF and pregnancy outcome; the expression of MSTN in the human endometrium and the regulation of endometrial cyclicity and its molecular mechanisms; the role of MSTN in placental insufficiency, gestational diabetes, preeclampsia, intrauterine growth restriction and other placenta-related disease pathology and potential therapeutic targets; changes in endocrine levels in MSTN-null individuals and implications for fertility. Developing a better understanding of MSTN's physiological roles in the human reproductive system will provide significant insights into pathology and will lead to new approaches to fertility regulation, whether the goal is to develop alternative methods of contraception, diagnose and treat human infertility, or develop safer and more reliable protocols for inducing ovulation in assisted reproductive technology (ART). Because MSTN signaling pathways are involved in a wide range of developmental and pathological events in reproductive biology, targeting these pathways as a therapeutic strategy for overcoming female infertility may be a viable treatment option. In the near future it may be possible to treat PCOS and OHSS by inhibiting the expression and function of MSTN in the ovary, but in the meantime it is important to note that inhibiting MSTN may cause increased muscle content, myofibrillar hyperplasia and decreased osteoclast numbers. As research continues, we will learn more about MSTN.

## Data Availability

The current study was based on results of relevant published studies.

## Abbreviations

MSTN: Myostatin
GDF8: Growth differentiation factor8
TGF-β: Transforming growth factor-β
OHSS: Ovary hyper-stimulation syndrome
PCOS: Polycystic ovarian syndrome
BMPs: Bone morphogenetic proteins
GDFs: Growth differentiation factors
AMH: Anti-Müllerian hormone
GCs: Granulosa cells
StAR: Steroidogenic acute regulatory
Aromatase: Cytochrome P450 aromatase
FSH: Follicle-stimulating hormone
LH: Luteinizing hormone
COC: Cumulus oocyte complex
COS: Controlled ovarian stimulation
ART: Assisted reproductive technologies
E2: Estradiol
CYP19A1: Cytochrome P450 Family 19 Subfamily A Member 1
hGL: Human granulosa-lutein
EVT: Extravillous trophoblast
HSD3B1: Hydroxy-Delta-5-Steroid Dehydrogenase, 3β-and Steroid Delta-Isomerase1
CTGF: Connective tissue growth factor
LOX: Lysyl oxidase
ECM: Extracellular matrix
PTX3: Pentraxin 3
P4: Progesterone
SMC: Smooth muscle cells
EEC: Endometrial epithelial cells
TM: Trophotoderm
hCG: Human chorionic gonadotrophin
FSTL3: Follistatin-like 3
PE: Pre-eclampsia
IUGR: Intrauterine growth restriction
GDM: Gestational diabetes mellitus
TNFα: Tumour necrosis factor α
